# Pan-cancer analysis of microRNA expression profiles highlights microRNAs enriched in normal body cells as effective suppressors of multiple tumor types: A study based on TCGA database

**DOI:** 10.1371/journal.pone.0267291

**Published:** 2022-04-27

**Authors:** Sharif Moradi, Aryan Kamal, Hamidreza Aboulkheyr Es, Farnoosh Farhadi, Marzieh Ebrahimi, Hamidreza Chitsaz, Ali Sharifi-Zarchi, Hossein Baharvand

**Affiliations:** 1 Cell Science Research Center, Department of Stem Cells and Developmental Biology, Royan Institute for Stem Cell Biology and Technology, ACECR, Tehran, Iran; 2 Center for Bioinformatics, Saarland University, Saarbrücken, Germany; 3 School of Biomedical Engineering, Faculty of Engineering and Information Technology, University of Technology Sydney, Sydney, NSW, Australia; 4 Computer Engineering Department, Sharif University of Technology, Tehran, Iran; 5 Department of Computer Science, Colorado State University, Fort Collins, CO, United States of America; 6 Department of Developmental Biology, University of Science and Culture, Tehran, Iran; Children’s National Hospital, UNITED STATES

## Abstract

**Background:**

MicroRNAs (miRNAs) are frequently deregulated in various types of cancer. While antisense oligonucleotides are used to block oncomiRs, delivery of tumour-suppressive miRNAs holds great potential as a potent anti-cancer strategy. Here, we aim to determine, and functionally analyse, miRNAs that are lowly expressed in various types of tumour but abundantly expressed in multiple normal tissues.

**Methods:**

The miRNA sequencing data of 14 cancer types were downloaded from the TCGA dataset. Significant differences in miRNA expression between tumor and normal samples were calculated using limma package (R programming). An adjusted *p* value < 0.05 was used to compare normal versus tumor miRNA expression profiles. The predicted gene targets were obtained using TargetScan, miRanda, and miRDB and then subjected to gene ontology analysis using Enrichr. Only GO terms with an adjusted *p* < 0.05 were considered statistically significant. All data from wet-lab experiments (cell viability assays and flow cytometry) were expressed as means ± SEM, and their differences were analyzed using GraphPad Prism software (Student’s *t* test, *p* < 0.05).

**Results:**

By compiling all publicly available miRNA profiling data from The Cancer Genome Atlas (TCGA) Pan-Cancer Project, we reveal a small set of tumour-suppressing miRNAs (which we designate as ’normomiRs’) that are highly expressed in 14 types of normal tissues but poorly expressed in corresponding tumour tissues. Interestingly, muscle-enriched miRNAs (*e*.*g*. miR-133a/b and miR-206) and miRNAs from *DLK1-DIO3* locus (*e*.*g*. miR-381 and miR-411) constitute a large fraction of the normomiRs. Moreover, we define that the CCCGU motif is absent in the oncomiRs’ seed sequences but present in a fraction of tumour-suppressive miRNAs. Finally, the gain of function of candidate normomiRs across several cancer cell types indicates that miR-206 and miR-381 exert the most potent inhibition on multiple cancer types *in vitro*.

**Conclusion:**

Our results reveal a pan-cancer set of tumour-suppressing miRNAs and highlight the potential of miRNA-replacement therapies for targeting multiple types of tumour.

## Introduction

MicroRNAs (miRNAs) are an extensive class of highly conserved, short regulatory RNAs which post-transcriptionally control gene expression in diverse eukaryotes and various cell types [1–3]. They are transcribed as primary miRNAs (pri-miRNAs) from specific loci of the genome mostly by RNA Pol II, and are therefore capped and poly-adenylated. The pri-miRNA, which can be thousands of nucleotides long, is then processed into a ~70-nucleotide precursor miRNA (pre-miRNA) either by spliceosome or microprocessor (a complex of DROSHA/DGCR8). The pre-miRNA is shuttled into the cytosol to undergo further processing by DICER/TRBP complex, yielding a mature double-stranded ~22-bp miRNA. One strand of this double-stranded RNA species is then preferentially selected by an Argonaute (AGO) protein which composes the main component of the so-called miRNA-induced silencing complex (miRISC). The mature single-stranded miRNA incorporated into miRISC will bind complementary sequences within target mRNAs, promoting their cleavage or translational repression. The net effect would be to block efficient translation of the target mRNAs into proteins [4].

Since miRNAs have a minimal binding requirement of only six consecutive nucleotides to pair with their target transcripts [5], they might have numerous mRNA targets, thereby enabling them to orchestrate complex gene expression programs and exert widespread effects on cell behaviour. Global miRNA activity is of crucial importance to embryonic development, as genetic ablation of key processing enzymes involved in miRNA maturation is embryonic lethal, highlighting the vital functions that are mediated by miRNAs in cellular and embryonic physiology [6, 7]. Importantly, loss of function of specific individual miRNAs has also been demonstrated to disrupt normal pre-, peri-, or post-natal development [8], further indicating essential roles played by these tiny RNAs in organismal development.

Abnormal expression and function of miRNAs have been linked to various diseases, including different cancers [9–17]. It has been shown that miRNA-coding sequences are frequently located in, or near, genomic regions associated with cancer, leading to aberrant activation or inactivation of miRNAs in diverse tumor types [18]. Global expression of miRNAs has been documented to be deregulated in most (if not all) cancers [19]. Functionally, a fraction of miRNAs promote tumorigenicity (also known as oncomiRs), while others suppress cancer cell growth and metastasis (also known as tumor-suppressive or anti-cancer miRNAs) [9, 14, 20, 21]. While antisense oligonucleotides are frequently used to prevent oncomiRs from functioning, delivery of tumor-suppressing miRNAs has emerged as a potent strategy to restrict tumor growth and metastasis [14]. It appears that the majority of miRNAs serve anti-cancer functions, as evidenced by enhanced growth and/or aggressiveness of tumors upon loss of function of DICER, DGCR8 or DROSHA [19]. In fact, many cancers downregulate global miRNA expression to facilitate their malignant transformation and tissue invasion. Kota and colleagues indicated that a normal tissue-enriched miRNA, namely miR-26a, can be used to effectively inhibit cancer cell growth both *in vitro* and in a mouse model of liver cancer [22]. Such miRNAs, which we designate as "normomiR" (i.e. showing high expression in multiple types of healthy tissues yet downregulated in various cancer types), are believed to be well tolerated by normal tissues of the body when administered systematically [22–26]. This is because normal tissues already express high levels of them (therefore, a further increase in their expression would not have notable adverse effects). We hypothesized that miRNAs that are poorly expressed in multiple cancer types but highly expressed in multiple corresponding normal tissues might be effective agents to target tumorigenesis of various cancers.

We investigated the global miRNA landscapes across 14 cancer types with their corresponding normal cell types to determine miRNAs that exhibit low expression in various tumors but abundant expression in normal tissues (i.e. identification of the so-called normomiRs). By compiling all publically available miRNA profiling data from the TCGA Pan-Cancer Project, which includes data from Prostate Adenocarcinoma (PRAD), Kidney Renal Clear Cell Carcinoma (KIRC or KIRH), Kidney Papillary Cell Carcinoma (KIRP), Kidney Chromophobe (KICH), Stomach Adenocarcinoma (STAD), Oesophageal Carcinoma (ESCA), Lung Squamous Cell Carcinoma (LUSC), Lung Adenocarcinoma (LUAD), Uterine Corpus Endometrial Carcinoma (UCEC), Bladder Urothelial Carcinoma (BLCA), Head and Neck Squamous Cell Carcinoma (HNSC), Thyroid Cancer (THCA), Breast Cancer (BRCA), and Liver Hepatocellular Carcinoma (LIHC), we revealed a small set of pan-cancer tumor-suppressing miRNAs that are highly expressed in various normal tissues while at the same time, poorly expressed in corresponding tumor types. Muscle-enriched miRNAs (also known as myomiRs) as well as miRNAs from the imprinted *DLK1-DIO3* locus constitute a large fraction of the final tumor-suppressive miRNAs. Interestingly, the CCCG motif appears to be absent in the oncomiRs’ seed sequences, but present in a fraction of final candidate anti-cancer miRNAs. Gain of function of nine of these potentially anti-cancer miRNAs across six cancer cell types indicates the miRNAs miR-206 and miR-381 exert the most powerful inhibition on multiple cancer types. Overall, our results reveal a pan-cancer set of tumor-suppressing miRNAs which effectively restrict tumor cell growth in vitro, and highlight the potential of miRNA replacement therapy for targeting multiple cancer types.

## Materials and methods

### Research design: The TCGA pan-cancer dataset analysis

The miRNA sequencing data of 14 cancer types as well as the corresponding normal cell types were downloaded from the TCGA pan-cancer dataset. We determined which miRNAs were consistently upregulated in normal cells (*i*.*e*. tumor-suppressing miRNAs or normomiRs) or in tumor cells (*i*.*e*. oncomiRs). Next, a seed sequence motif analysis was performed to examine if specific nucleotide motifs might define oncomiRs or normomiRs. We also asked whether miRNAs enriched in ’specific’ normal tissues might contribute more commonly to the final set of candidate normomiRs. Next, we sought to shortlist the normomiRs in order to perform functional analyzes and find the best miRNA candidates capable of simultaneously inhibiting multiple cancer cell types. The most potent miRNAs were then subjected to in silico analyses to identify their putative targets and define major cancer-related regulatory processes on which these miRNAs could exert inhibitory effects.

### Acquisition of TCGA data

The TCGA miRNA profiling data were downloaded directly from the TCGA data portal of TCGA Pan-Cancer project (https://tcga-data.nci.nih.gov/tcga/). This project consists of 14 available tumor types that include PRAD, KIRH, KIRP, KICH, STAD, ESCA, LUSC, LUAD, UCEC, BLCA, HNSC, THCA, BRCA, and LIHC. Multiple reads from individual miRNA isoforms were compiled into a single read count; we used the reads per million miRNAs mapped data form, in which each miRNA read count is established as a fraction of the total miRNA population. miRNA profiling data were normalized using the quantile normalization from limma package. The significant differences in miRNA expression between tumor and normal samples were calculated using limma package with Benjamini-Hochberg correction to determine false discovery rates (FDRs).

### Determination of pan-cancer pro-tumor and anti-tumor miRNAs

To determine miRNAs that exhibit consistent expression changes across the majority of tumor types tested, a stringent threshold (adjusted *p* value < 0.05, with Benjamini-Hochberg correction for multiple testing) was used to compare normal versus tumor miRNA expression profiles. Our threshold required pan-cancer tumor-suppressing miRNAs to be significantly upregulated in 12 out of 14 cancer types with available normal versus cancer data. On the other hand, pan-cancer oncomiRs were defined to have reduced expression in 12 out of 14 cancer types.

### Prediction of miRNA target and GO analyzes

TargetScan (www.targetscan.org) [27], miRanda (www.microrna.org) [28], and miRDB (www.midb.org) [29] databases were used to obtain the list of predicted mRNA targets of miRNAs. The predicted gene targets were then subjected to gene ontology (GO) Biological Process analysis using Enrichr (http://amp.pharm.mssm.edu/Enrichr/) [30]. To identify oncogenes potentially targeted by normomiRs, we intersected the predicted targets of all the normomiRs (obtained using TargetScan) with all the experimentally validated oncogenes, obtained from CancerMine (http://bionlp.bcgsc.ca/cancermine) [31], that have previously been identified in various types of cancer. Only GO terms with an adjusted P<0.05 were considered statistically significant and represented.

### Cell culture

Cancer cells (1.5–3.0 × 10^3^ cells/well) were cultured on tissue-culture 96-well plates (Sigma-Aldrich) in Knockout™ DMEM (Invitrogen) supplemented with 15% fetal bovine serum (HyClone), 0.1 mM β-mercaptoethanol (Sigma-Aldrich), 100 U/ml penicillin, 100 μg/ml streptomycin (Invitrogen), and 2 mM L-glutamine (Invitrogen), and passaged every other day.

### Viability assays

After removal of the culture media, the MTS reagent (Promega) was directly added to the wells in 96-well plates, and the cells were then maintained in a 37°C incubator for 1–3 hours. Cell viability measurements were performed by determining absorbance at 495 nm on a Multiskan MCC microplate reader (Thermo Fisher Scientific).

### Transient transfection of miRNAs

Cells were transfected with 100 nM of each miRNA mimic (Dharmacon, miRIDIAN microRNA mimics, Thermo Fisher Scientific) according to the manufacturer’s instructions. The scrambled small RNA control (Scr) or the candidate miRNA mimics as well as the DharmaFECT1 transfection reagent (Dharmacon, Thermo Fisher Scientific) were diluted in serum-free DMEM/F-12, mixed, and incubated for 20 minutes at room temperature. DharmaFECT1-small RNA complexes were added to the culture media in a drop-wise manner. To determine the efficiency of small RNA transfection into cancer cells, we utilized FITC-labelled small RNA to transfect the cells. Twenty four hours post-transfection, flow cytometry was used to assess the percentage of cells positive for FITC. Flow cytometry of the cells transfected with FITC-conjugated small RNA was performed using a BD LSR II flow cytometer (BD Biosciences) and the data were analyzed using BD FACSDiva (BD Biosciences). Assays were performed with three biological replicates and the data are represented as the mean ± SEM.

### Statistical analysis

All data from wet-lab experiments are expressed as means ± SEM. GraphPad Prism software (GraphPad Software, Inc., La Jolla, CA) was used to analyze the differences using Student’s *t* test, and a P<0.05 was considered statistically significant.

## Results and discussion

### Identification of pan-cancer normomiRs

**Fig 1:**
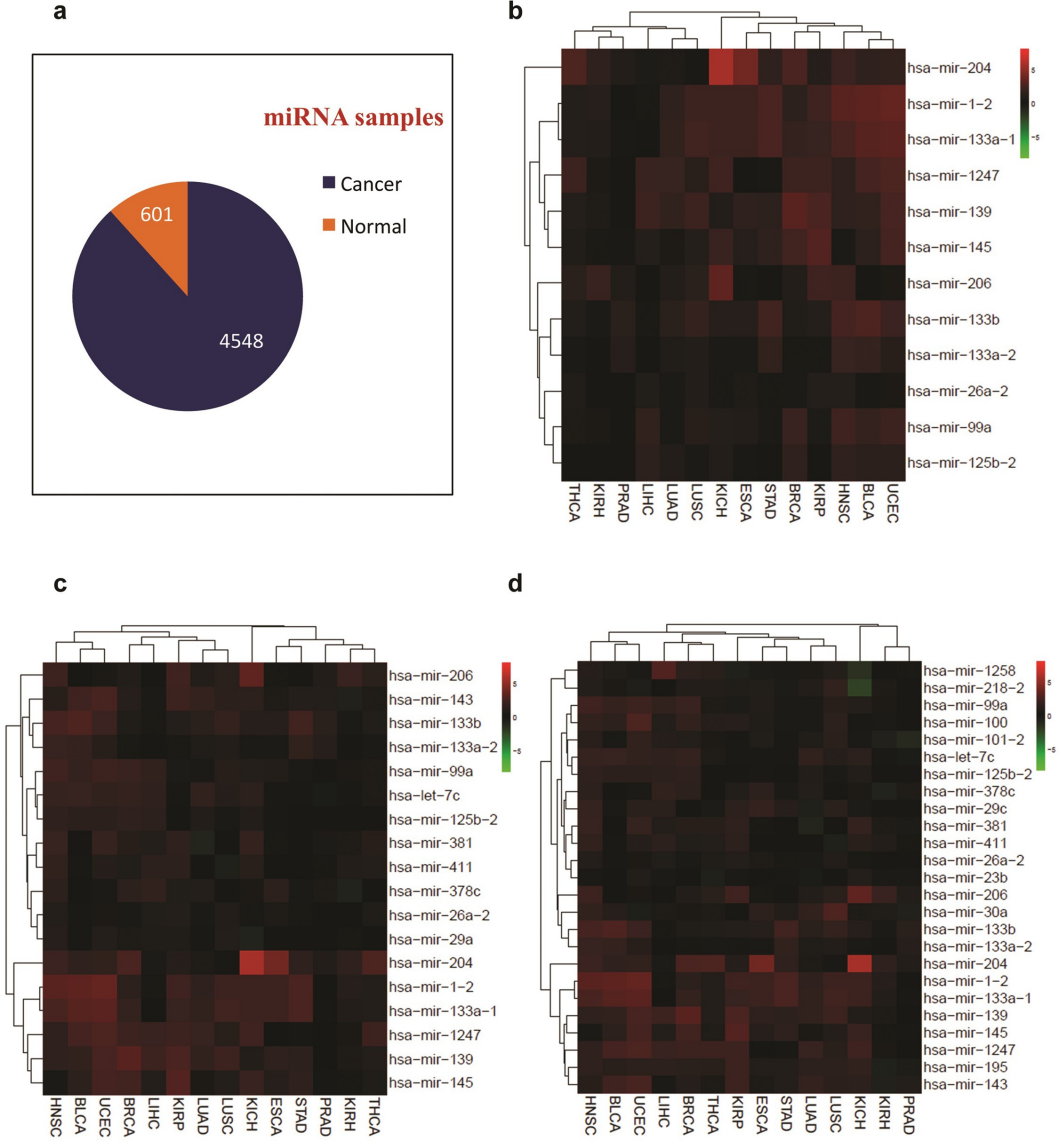
Identification of pan-cancer normomiRs. (**a**). The total number of global miRNA expression profiles from normal and tumor cells. (**b**). Heatmap of miRNAs showing higher expression in 14 out of 14 normal tissue types than corresponding tumor cells. (**c**). The miRNAs showing higher expression in 13 out of 14 normal tissue types than corresponding tumor cells. (**d**). The miRNAs with higher expression in 12 out of 14 normal tissue types than corresponding cancer cell types.

First, we wanted to determine top normal tissue-upregulated miRNAs across multiple types of normal cells in comparison to corresponding tumor samples. The downloaded data from the TCGA dataset included 14 major types of cancers which included KICH, STAD, OSCA, PRAD, KIRH, KIRP, BLCA, HNSC, THCA, LUSC, LUAD, LIHC, BRCA, and UCEC. Of note, we obtained a much larger number of samples for tumors versus normal samples (Fig 1A), which was expected, as the TCGA dataset mainly contains omics data (including global miRNA expression profiles) of tumor samples. We attempted to find miRNAs which exhibit higher levels of expression in 14 out of 14 normal tissues than corresponding tumor samples. This analysis was revealed to be too stringent, as very few miRNAs could meet this criterion (Fig 1B). We, therefore, reduced the stringency and found that a minimum 12 out of 14 level of stringency yields a 25-miRNA list of pan-cancer normomiRs (Fig 1C and 1D). The normomiRs identified by our analysis include, but are not limited to, miR-145, let-7c, miR-26a, miR-381, miR-1, and miR-206 (Fig 1D). Importantly, the majority of these candidate miRNAs have previously been reported to inhibit the growth and/or invasiveness of cancer cells [22, 32–34], indicating that our analysis of normomiRs yields reliable anti-cancer miRNAs with pan-cancer inhibitory potential. Notably, our analysis also yielded some normomiRs, e.g. miR-139 and miR-195, which have not been broadly investigated in multiple cancer types, suggesting potentially anti-cancer miRNAs with pan-cancer effects which need to be further functionally characterized. We then sought to determine the oncogenes that could be targeted by the normomiRs in various cancer types. To this end, we obtained the predicted targets of normomiRs using TargetScan and intersected them with the list of experimentally verified oncogenes reported in the CancerMine database. This analysis indicated that a large number of oncogenes were targeted by normomiRs (S1 Table), which highlights the potential anti-cancer functions of normomiRs. Notably, we found that 27 of these oncogenes were co-targeted by at least five of the normomiRs (Table 1), which suggests that the potential pan-cancer inhibitory effects of normomiRs may stem from their ability to target multiple oncogenes simultaneously. Overall, we provide a short list of normomiRs with potentially anti-cancer effects across various types of cancer.

**Table 1:** The oncogenes predicted to be co-targeted by several normomiRs in various cancer types.

Gene symbol	Regulating normomiRs (#)	Regulating normomiRs (miRNA IDs)
TAOK1	10	hsa-miR-99a-5p, hsa-miR-30a-5p, hsa-miR-100-5p, hsa-miR-26a-5p, hsa-miR-195-5p, hsa-miR-133b, hsa-miR-139-5p, hsa-miR-23b-3p, hsa-miR-204-5p, hsa-miR-145-5p
G3BP1	7	hsa-miR-30a-5p, hsa-miR-125b-5p, hsa-miR-1-3p, hsa-miR-145-5p, hsa-miR-204-5p, hsa-miR-206, hsa-let-7c-5p
LASP1	7	hsa-miR-218-5p, hsa-miR-29c-3p, hsa-miR-206, hsa-miR-145-5p, hsa-miR-23b-3p, hsa-miR-133b, hsa-miR-1-3p
TET3	7	hsa-miR-381-3p, hsa-miR-29c-3p, hsa-miR-26a-5p, hsa-miR-23b-3p, hsa-miR-133b, hsa-miR-139-5p, hsa-let-7c-5p
DNMT3A	6	hsa-miR-145-5p, hsa-miR-29c-3p, hsa-miR-26a-5p, hsa-miR-30a-5p, hsa-miR-1-3p, hsa-miR-206
FUBP1	6	hsa-miR-218-5p, hsa-miR-30a-5p, hsa-miR-26a-5p, hsa-miR-195-5p, hsa-miR-206, hsa-miR-1-3p
RORA	6	hsa-miR-29c-3p, hsa-miR-30a-5p, hsa-miR-206, hsa-miR-1-3p, hsa-miR-23b-3p, hsa-miR-125b-5p
SEMA6D	6	hsa-miR-206, hsa-miR-1-3p, hsa-miR-26a-5p, hsa-miR-23b-3p, hsa-miR-30a-5p, hsa-miR-195-5p
A1CF	5	hsa-miR-26a-5p, hsa-miR-195-5p, hsa-miR-143-3p, hsa-let-7c-5p, hsa-miR-30a-5p
CBL	5	hsa-miR-206, hsa-miR-143-3p, hsa-miR-378c, hsa-miR-1-3p, hsa-let-7c-5p
CCDC6	5	hsa-miR-26a-5p, hsa-miR-23b-3p, hsa-miR-30a-5p, hsa-miR-218-5p, hsa-miR-195-5p
CDK6	5	hsa-miR-26a-5p, hsa-miR-145-5p, hsa-miR-1-3p, hsa-miR-206, hsa-miR-218-5p
FGFR3	5	hsa-miR-100-5p, hsa-miR-1-3p, hsa-miR-206, hsa-miR-99a-5p, hsa-miR-23b-3p
FOSL2	5	hsa-miR-30a-5p, hsa-miR-195-5p, hsa-miR-218-5p, hsa-miR-143-3p, hsa-miR-125b-5p
GAN	5	hsa-miR-30a-5p, hsa-miR-206, hsa-miR-1-3p, hsa-miR-26a-5p, hsa-let-7c-5p
IGF1	5	hsa-miR-29c-3p, hsa-miR-206, hsa-miR-1-3p, hsa-miR-378c, hsa-let-7c-5p
KLF12	5	hsa-miR-381-3p, hsa-miR-29c-3p, hsa-miR-218-5p, hsa-miR-145-5p, hsa-miR-30a-5p
KLF13	5	hsa-miR-143-3p, hsa-miR-1-3p, hsa-miR-206, hsa-miR-30a-5p, hsa-miR-125b-5p
OTUD4	5	hsa-miR-26a-5p, hsa-miR-29c-3p, hsa-miR-195-5p, hsa-miR-143-3p, hsa-miR-23b-3p
PDE7A	5	hsa-miR-1-3p, hsa-miR-23b-3p, hsa-miR-30a-5p, hsa-miR-206, hsa-miR-218-5p
RCOR1	5	hsa-miR-26a-5p, hsa-miR-23b-3p, hsa-miR-30a-5p, hsa-miR-218-5p, hsa-miR-204-5p
RFX3	5	hsa-miR-218-5p, hsa-miR-195-5p, hsa-miR-30a-5p, hsa-miR-145-5p, hsa-miR-133b
ROBO1	5	hsa-miR-23b-3p, hsa-miR-218-5p, hsa-miR-29c-3p, hsa-miR-139-5p, hsa-let-7c-5p
SLC7A11	5	hsa-miR-26a-5p, hsa-miR-206, hsa-miR-30a-5p, hsa-miR-1-3p, hsa-miR-143-3p
TPM3	5	hsa-miR-143-3p, hsa-miR-1-3p, hsa-miR-145-5p, hsa-miR-206, hsa-miR-204-5p
ZBTB20	5	hsa-miR-204-5p, hsa-miR-195-5p, hsa-miR-139-5p, hsa-miR-378c, hsa-miR-143-3p
ZBTB7A	5	hsa-miR-204-5p, hsa-miR-30a-5p, hsa-miR-206, hsa-miR-1-3p, hsa-miR-125b-5p

### miRNAs highly expressed in various types of cancers

Hamilton et al. [35] previously reported a list of pan-cancer oncomiRs which displayed enhanced expression in at least 6 out of 8 tumor samples versus corresponding normal cells. We sought to determine which oncomiRs were expressed higher in at least 12 out of 14 tumor types compared with normal cells (i.e. twice as many cancer types as used in Hamilton et al. study), thereby providing a more reliable pan-cancer list of oncomiRs. Our analysis yielded 25 miRNAs which were more abundantly expressed in tumor cells than normal tissues (S1A Fig). We observed a significant overlap with data reported by Hamilton et al. in terms of the miRNAs exhibiting higher expression levels in multiple cancer cells. For example, miRNAs such as miR-21, miR-17, miR-93, miR-19a, and miR-130b were observed to be the most consistently upregulated miRNAs in various tumor samples (S1A and S1B Fig), as reported previously [35].

In addition to these commonly identified miRNAs, we found yet other oncomiRs including miR-181, miR-1307, miR-1301, miR-20a, miR-155, miR-106b, miR-15a, miR-16, miR-629, miR-454, miR-937, miR-3127, miR-769, miR-671, and miR-589 to be abundantly expressed in the majority of cancer types analyzed as compared to normal tissues (S2A and S2B Fig). Furthermore, some of the oncomiRs identified as pan-cancer oncomiRs in Hamilton et al. study (i.e. miR-210, miR-106a, miR-135b, miR-301b, miR-192, miR-142, miR-301a, miR-33b, miR-590, miR-196a, miR-7, and miR-455) were not included in our final list of oncomiRs, probably due to the inclusion of a lower number of cancer types in the aforementioned study and the higher stringency applied in our analysis. Finally, we looked at the previously reported GUGC motif in oncomiRs identified by Hamilton et al. and observed that eight of our identified oncomiRs had this typical oncomiR-associated nucleotide motif in their seed sequence.

The identification of a more or less similar set of oncomiRs in both studies further highlights the reliability of our analysis. Although there was a fraction of pan-cancer oncomiRs that were differentially identified in the two studies, the fact that these miRNAs belong to some of the same miRNA families underscores the high similarity of the final set of oncomiRs between the Hamilton et al. and our study. Importantly, almost all of the oncomiRs that we identified across multiple tumor types are known to be upregulated, and functionally important, in pluripotent stem cells such as embryonic stem cells and induced pluripotent stem cells [36–38]. These stem cells are characterized by unlimited self-renewal property and can form benign teratomas or malignant teratocarcinomas upon direct transplantation into immunocompromised mice [39, 40], suggesting that the identified oncomiRs might facilitate the malignant transformation as well as invasiveness of various cancer cells by promoting stemness characteristics in emerging tumor cells.

### Specific short sequence motifs define a fraction of normomiRs

**Fig 2:**
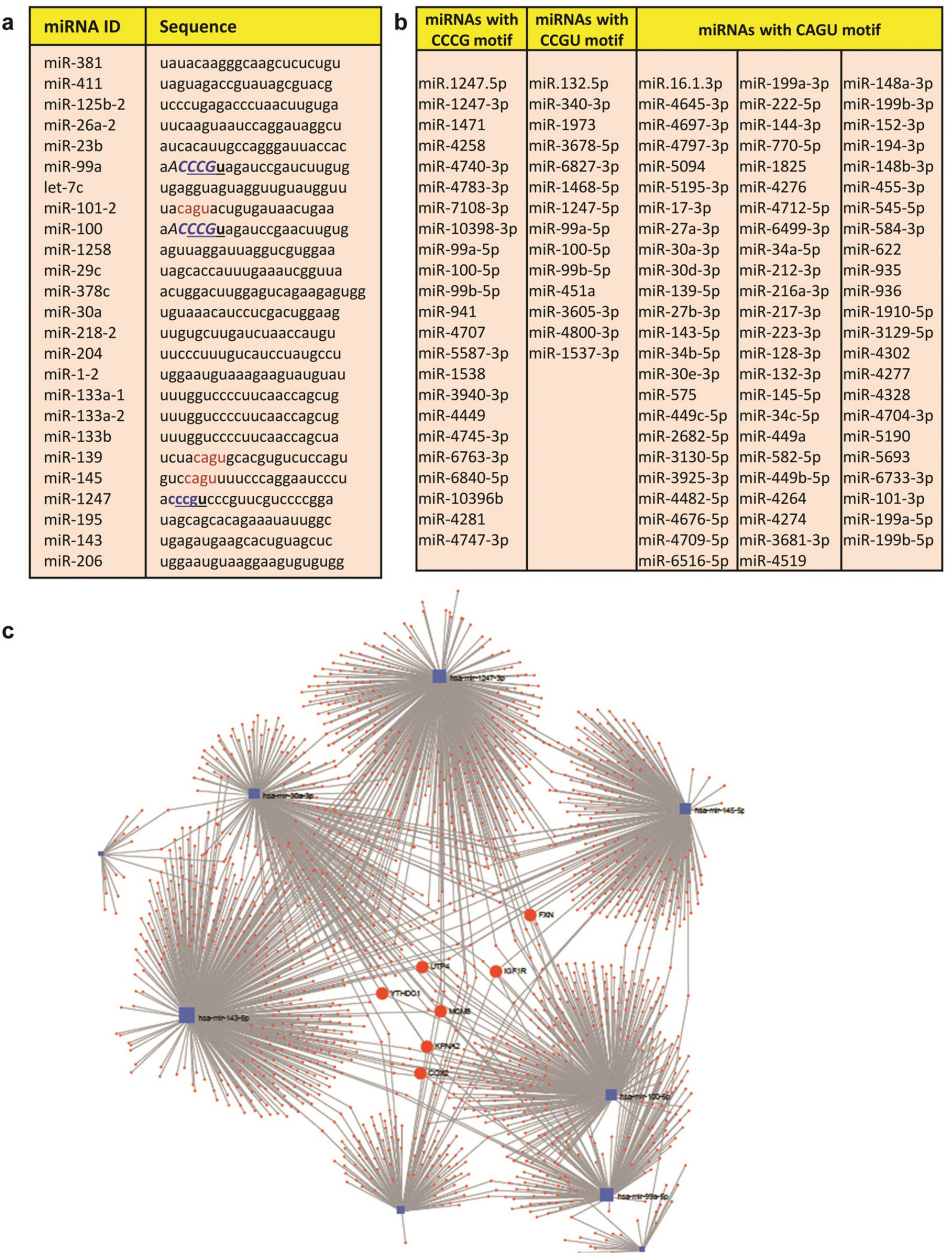
A fraction of tumor-suppressive miRNAs are characterized by specific short nucleotide motifs. (**a**). The frequency of certain four- and five-nucleotide motifs in the seed regions of normomiRs: the CCCG motif (blue) was found in miR-100, miR-99a, and miR-1247, the CCGU motif (underlined sequence) was present in in the same three normomiRs, the CAGU motif (red) was found in miR-101, miR-139, and miR-145, the CCCGU motif (**bold**) was present in miR-99a, -100, and -1247, and the ACCCG motif (*italic*) was present in miR-99a and miR-100. (**b**). The list of all miRNAs across human miRNome containing the CCCG, CCGU, and/or CAGU motifs in their seed regions. (**c**). Identification of the mRNA-miRNA network of the normomiRs containing the three sequence motifs CCCG, CCGU, and CAGU. These normomiRs engage in a highly dynamic regulatory circuitry in which many cancer-critical genes (particularly *FXN*, *IGF1R*, *COX2*, *UTP4*, *YTHDC1*, *MCM8*, and *KPNA2*) are co-regulated.

We next asked if we can similarly find a common four-nucleotide motif within the seed sequences (nucleotides 2–8 from 5’ terminus) of the identified normomiRs. Our motif analysis found a four-nucleotide sequence, i.e. the CCCG motif, which was observed in three of the normomiRs: miR-100, miR-99a, and miR-1247 (Fig 2A). Intriguingly, none of the 25 pan-cancer oncomiRs identified in our study contained the CCCG motif (S1B Fig), making this short sequence motif depleted among the identified oncomiRs. This four-nucleotide sequence might be a key determinant in defining the range of target mRNAs commonly regulated by the CCCG-containing normomiRs. This seed motif similarity in a fraction of pan-cancer normomiRs suggests that these miRNAs might undergo coordinate regulation to commonly target crucial oncogenes. We next examined the frequency of CCCG motif across all human miRNAs and not just the miRNAs from our final candidates. This analysis yielded 256 four-nucleotide motifs across the human miRNome (S2A Fig and S2 Table). We observed that a total of 23 miRNAs contain the CCCG motif in their seed sequence (Figs 2B and S2A and S2 Table) and that this motif was found in four miRNAs (including the three normomiRs above) with anti-cancer functions but was completely absent in known oncomiRs (Figs 2B and S1B and S2A).

Notably, our comprehensive analysis also yielded two other four-nucleotide motifs within the human miRNome which were more prevalent in miRNAs with potential anti-cancer functions: CCGU and CAGU (S2A Fig). The CCGU motif was present in 14 miRNA seed sequences, four of them exhibiting tumor suppressing activities. This motif was present in three of our 25 final normomiRs (overlapping with the CCCG motif) but again absent in known oncomiRs. The other motif, CAGU, was found in the seed regions of 71 miRNAs totally, with four of them exhibiting anti-tumor and one with pro-tumor functions. Three of our normomiRs (miR-101, miR-139, and miR-145) contained the CAGU motif in their seed region (Figs 2A and S2A). We also looked at the frequency of the five-nucleotide motifs in the seed region of all human miRNAs. Our results indicated that the CCCGU motif (containing the four-nucleotide CCCG motif mentioned before) was present in only four miRNAs, three of them were among our normomiRs (miR-99a, -100, and -1247), but depleted from oncomiRs (Fig 2A and S3 Table and S2B Fig). The miRNAs miR-99a and miR-100 also contained another five-nucleotide motif, ACCCG (containing the four-nucleotide ACCC motif), which was again depleted from oncomiRs (Figs 2A and S2B).

Next, to examine how a common seed motif (CCCG) might provide the miRNAs with this motif with a shared set of target genes potentially involved in tumorigenesis, we obtained the predicted list of transcripts co-targeted by of all these miRNAs from TargetScan, miRDB, and miRanda databases (S4 Table). Interestingly, our analysis suggested that the three CCCG-containing miRNAs could inhibit crucial cancer-associated pathways involved in cell cycling, glioma, melanoma, prostate cancer progression, and HIF-1 signaling (S2C Fig). We finally sought to identify the mRNA-miRNA network of the normomiRs containing the three sequence motifs CCCG, CCGU, and CAGU to examine if they converge on a small set of genes potentially implicated in cancer promotion. As shown in Fig 2C, these miRNAs engage in a highly interactive regulatory network in which they regulate many genes. Importantly, three of the normomiRs containing the above-mentioned motifs could co-target seven genes simultaneously. These genes (*FXN*, *IGF1R*, *COX2*, *UTP4*, *YTHDC1*, *MCM8*, and *KPNA2*) might be interesting targets for suppression in various cancers, as some of them (e.g. *COX2* and *IGF1R*) are already known to be potent drivers of tumorigenesis [41, 42]. Taken together, we uncover a number of previously uncharacterized short sequence motifs, enriched in the seed region of normomiRs but depleted in oncomiRs, which might mediate the suppression of various types of cancer and inhibit some of the biological processes driving malignant transformation of the cells.

### MyomiRs as well as miRNAs from *DLK1-DIO3* locus might resist tumorigenesis

**Fig 3:**
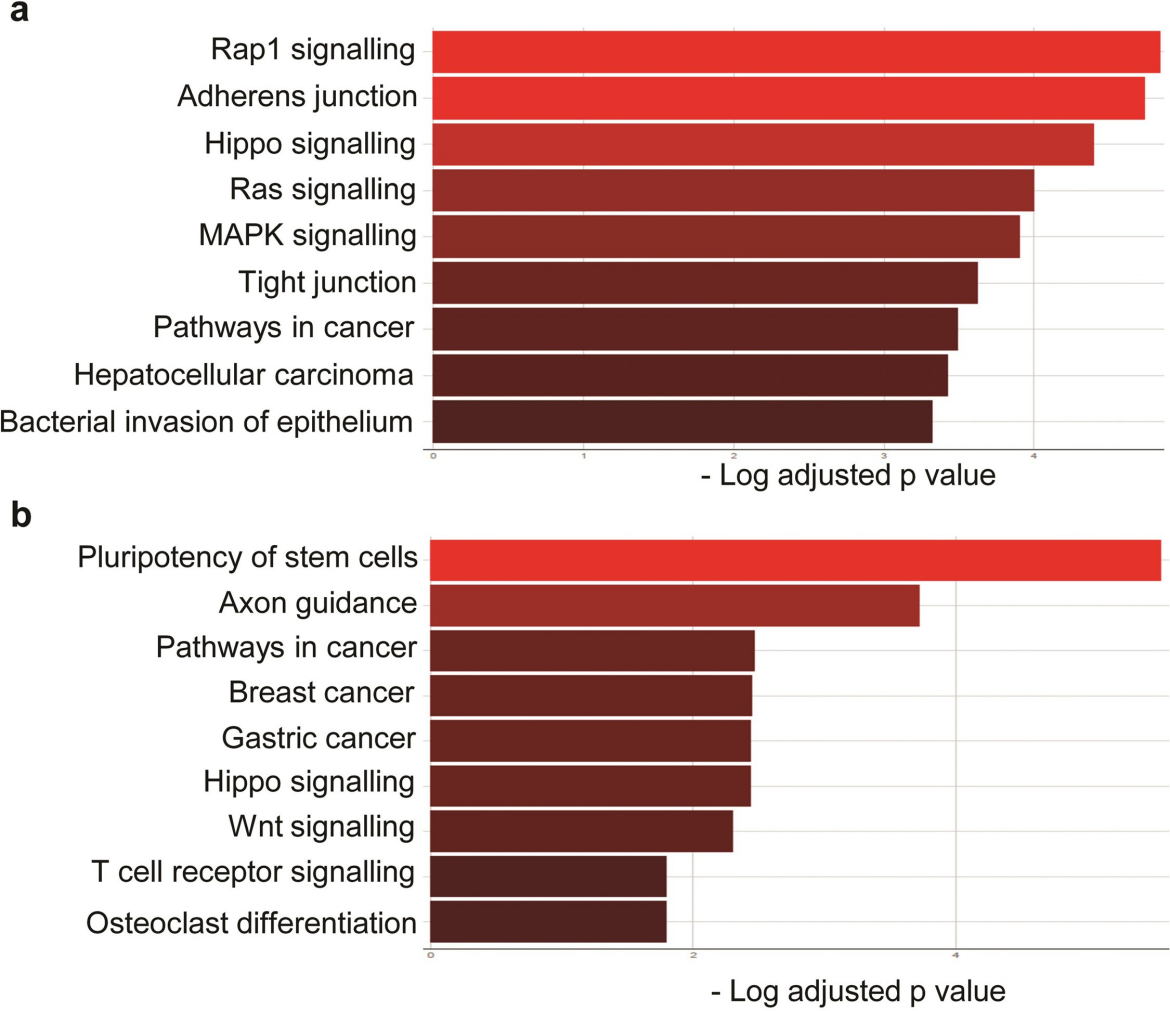
MyomiRs and certain miRNAs from *DLK1-DIO3* locus constitute a fraction of normomiRs. (**a**). KEGG analysis of genes predicted to be targeted by myomiRs. The gene targets of myomiRs were predicted using TargetScan and the GO analysis was performed using the KEGG feature of Enrichr. (**b**). GO analysis of genes predicted by TargetScan to be targeted by miR-381, miR-411, and miR-1247. These miRNAs belong to the *DLK1-DIO3* locus-embedded miRNA cluster.

Next, we sought to determine if the identified normomiRs might be representing certain genomic regions and/or belonging to specific miRNA families or clusters. We noticed that several of our identified normomiRs belong to the muscle-enriched family of miRNAs known as myomiRs which serve anti-tumor functions: miR-133a, miR-133b, miR-206, and miR-1 [34, 43, 44]. Notably, a large fraction of other miRNAs in the list including miR-100, miR-145, miR-195, miR-139, miR-381, and miR-411 are reported to be expressed in muscle cells and promote myogenic differentiation [43, 45–47]. The overrepresentation of muscle-associated miRNAs in our analysis is highly interesting, as muscle is among the few body tissues which do not (or only rarely) develop cancer, suggesting that these miRNAs might be among the key factors naturally resisting cancer development in muscle tissue and that they could be exploited for fighting multiple cancer types. Although these miRNAs are abundantly expressed in muscle, we found that they exhibited an enhanced expression level in other normal tissues than the majority of tumor samples analyzed (Fig 1D). To gain insight into the potential biological processes regulated by these miRNAs, we performed miRNA target prediction analysis using TargetScan and subjected the obtained targets (S5 Table) to gene ontology (GO) analysis. Results of KEGG analysis strongly suggested that these myomiRs could frequently target critical oncogenic pathways such as MAPK/Ras signaling and converge on a shared set of genes promoting the stemness and malignant transformation of tumor cells (Fig 3A).

Apart from myomiRs, we also noticed the enhanced expression of three miRNAs miR-381, miR-1247, and miR-411 in various normal cell types compared with corresponding tumor cells (Fig 1D). These miRNAs are expressed from the imprinted *DLK1-DIO3* locus at 14q32, encoding miR-379/410 mega cluster, the largest known miRNA locus in placental mammals. This locus codes for a large number of long and small non-coding RNAs including tens of miRNAs with diverse functions including in stem cell regulation, normal development, and diseases including cancer [48–50]. Importantly, *DLK1-DIO3* miRNAs tend to serve tumor-suppressing functions in many cancer types [51–53]. We, therefore, investigated *in silico* the potential gene networks that are regulated by these miRNAs to find out which pathways are potentially regulated by these miRNAs. Our analysis suggested that these miRNAs could target several genes (S6 Table) and pathways such as Wnt signaling critically involved in the development and progression of several types of cancer (Fig 3B). Overall, these findings suggest that miRNAs associated with muscle differentiation and the *DLK1-DIO3* miRNAs might provide natural barriers to tumorigenesis and that these miRNAs might be a viable option to target several cancer types simultaneously.

### miR-206 and miR-381 exhibit pan-cancer tumor-suppressing effects

In the next step, we wanted to examine which candidate miRNAs might simultaneously inhibit multiple tumor types *in vitro*. First, we tried to further narrow down the number of final candidate miRNAs for functional analysis. We chose all of the myomiRs except miR-133a, since it is almost identical to another selected myomiR, i.e. miR-133b (these two miRNAs differ from each other in only a single nucleotide located outside of the seed region. We also selected two of the CCCG-motif-containing miRNAs, i.e. miR-100 and miR-1247. miR-145 and miR-381 were also chosen because several separate studies report that they might inhibit multiple tumor types when overexpressed [54–56]. Of note, miR-1247 and miR-381 belong to the *DLK1-DIO3* locus-embedded miRNA cluster, as described above. Finally, we also included miR-195 and miR-139 for functional analysis, as they had been poorly studied in the context of tumorigenesis, and we wanted to further investigate their potential anti-cancer impacts on various cancer cell types *in vitro*.

**Fig 4:**
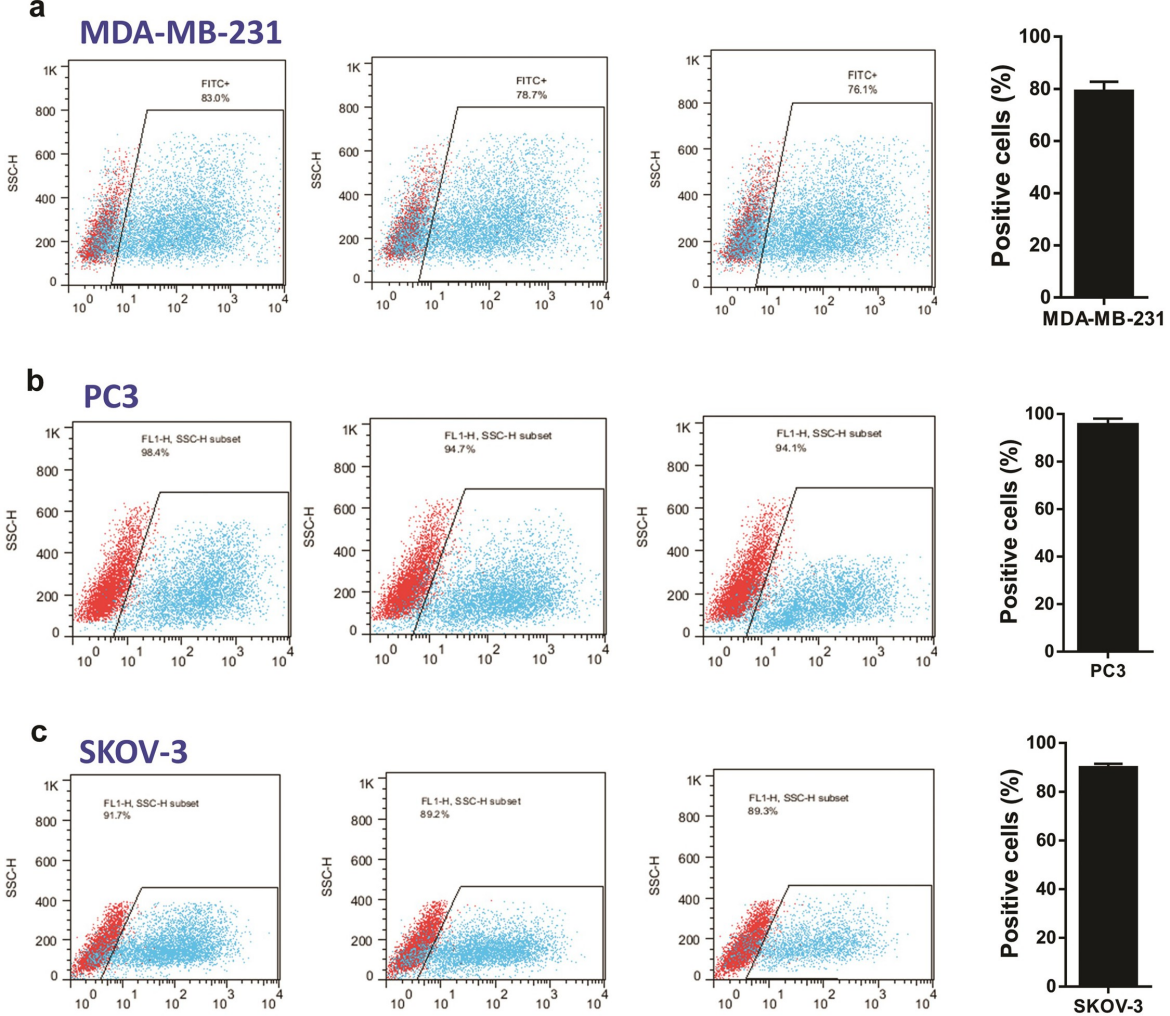
The efficiency of small RNA delivery into cancer cells. The MDA-MB-231 breast cancer cell line (**a**), PC3 prostate cancer cell line (**b**), and SKOV-3 ovarian cancer cell line (**c**) were treated with FITC-labelled small RNA and the cells were subjected to flow cytometry 24 hours post-transfection. The bar plots on the right show the mean calculated efficiency of small RNA delivery in percentage.

The cancer cell lines which were chosen for miRNA gain of function studies included LnCAP (a prostate cancer cell line with mesenchymal phenotype), PC3 (a prostate cancer cell line with epithelial phenotype), MDA-MB-231 (a malignant/metastatic breast cancer cell line with mesenchymal phenotype), A549 (a lung cancer cell line with mesenchymal phenotype), Huh-7 (a liver cancer cell line with epithelial phenotype), and SKOV3 (an ovarian cancer cell line with mesenchymal phenotype). These cancer types are already known to be either highly prevalent worldwide and/or among the deadliest cancers reported to date [57, 58]. Before cancer cell treatment with candidate mature miRNA mimics, we analyzed the efficiency of small RNA delivery into three types of cancer cell line (MDA-MB-231 breast cancer cells, PC3 prostate cancer cells, and SKOV-3 ovarian cancer cells) using FITC-conjugated small RNAs by transient transfection. Our flow cytometry analysis indicated that FITC-labelled small RNA could be delivered to different cancer cell lines with very high efficiency (from 76.1% to 98.4% of the cells for different cells) 24 hours post-transfection (Fig 4A–4C).

**Fig 5:**
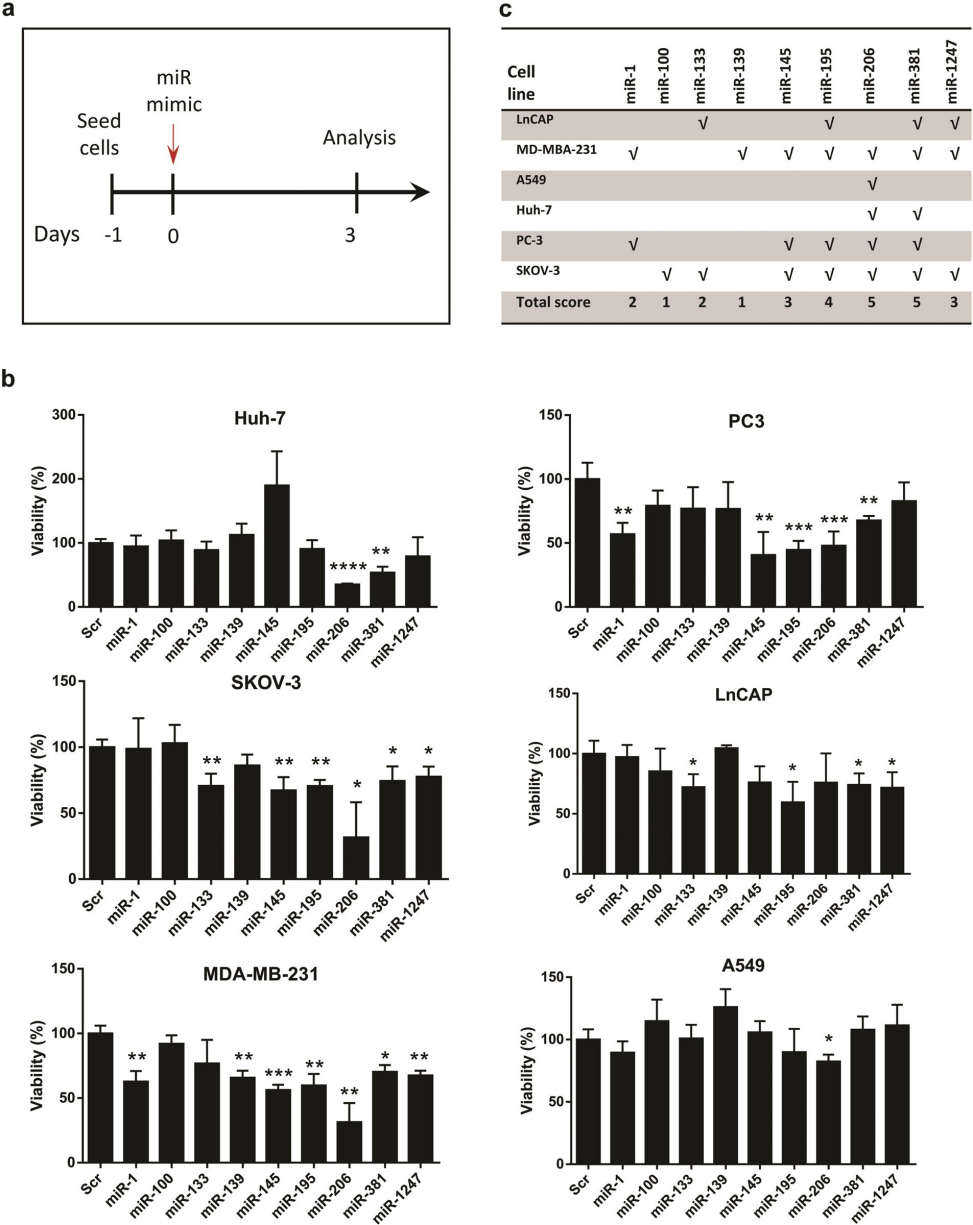
Gain of function of nine candidate normomiRs in six cancer cell types. (**a**). Schematic showing the procedure of cancer cell analysis following treatment with candidate normomiRs. (**b**). MTS viability assays of different cancer cell lines three days after transfection with candidate normomiRs. Data are shown as mean ± SEM, n = 3–5, *p<0.05; **p<0.001; ***p<0.0001; ****p<0.0001. (**c**). Summary of the results shown in (B). Huh-7: human hepatoma (hepatocellular carcinoma) cell line; PC3: prostate cancer 3 cell line, SKOV-3: an ovarian cancer cell line originally derived from ascites of a female patient with ovarian cancer; LnCAP: a prostate cancer cell line originally established from a metastatic lymph node lesion of prostate cancer; MDA-MB-231: a breast cancer cell line initially derived from a pleural effusion of a breast cancer patient with ductal carcinoma; A549: a lung cancer cell line isolated from a cancerous lung tissue in the explanted tumor tissue of a man with pulmonary adenocarcinoma.

For functional analysis of candidate miRNAs, cancer cells were seeded 24 hours prior to miRNA transfection and were subjected to viability assessment three days post-treatment (Fig 5A). Interestingly, we observed that except miR-139 and miR-100 whose overexpression inhibited only one cancer cell line (MDA-MB-231 and SKOV3, respectively), all the other candidate normomiRs significantly suppressed the growth of at least two cancer cell types (Fig 5A and 5B). We also found that miR-145 which is reported to inhibit various cancer cell types, could, in our hands, inhibit the growth of three of the six cancer cell types tested. Most importantly, it was revealed that two of the nine candidate miRNAs analyzed, i.e. miR-206 and miR-381, were the most potent pan-cancer normomiRs in suppressing multiple tumor types, since they considerably decreased the viability of five out of the six cancer types: the myomiR miR-206 could suppress all cancer types but LnCAP prostate cancer cell line, and the *DLK1-DIO3*–embedded miRNA miR-381 inhibited all but A549 lung cancer cells (Fig 5B and 5C). Therefore, miRNAs miR-381 and miR-206 can be powerful tools to suppress various cancer cell types. Notably, these two miRNAs did not exert any negative influence on the growth of human dermal fibroblast cells (S3 Fig), suggesting that their growth-inhibitory effects are restricted to cancer cells. Overall, our analyses revealed specific normomiRs with high potential to be investigated in future miRNA replacement therapies against multiple types of tumor cells in animal models and human clinical trials of cancer.

### Gene targets of miR-206 and miR-381 are critically involved in tumor development

**Fig 6:**
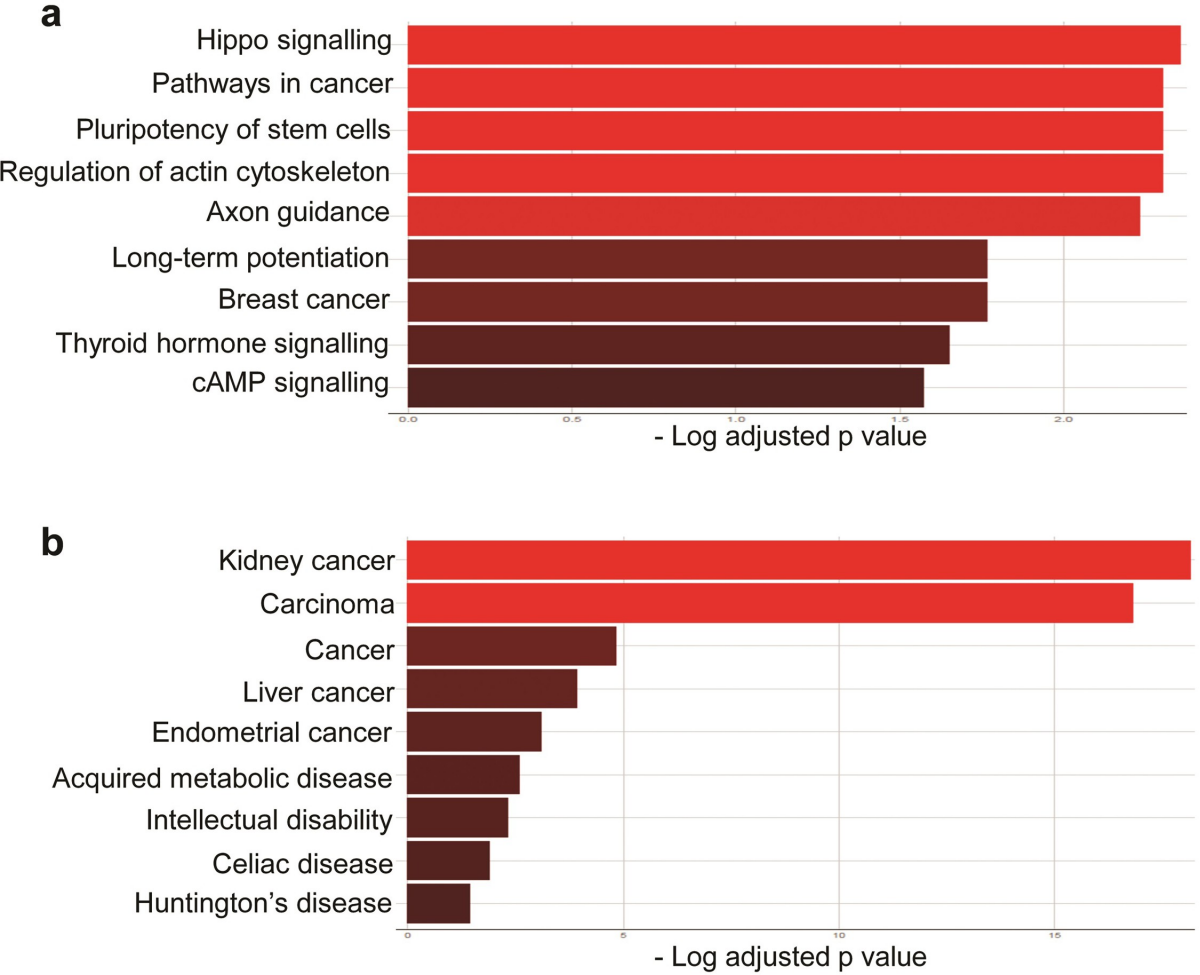
Potential biological processes regulated by miR-206 and miR-381. (**a**). Enrichr-based KEGG analysis of genes predicted to be co-targeted by miR-206 and miR-381. (**b**). The JENSEN Disease analysis of genes potentially co-regulated by miR-381 and miR-206. For both analyzes, the predicted gene targets of these miRNAs were obtained using TargetScan.

Having specified the most potent normomiRs against multiple cancer cell types, we next sought to analyze the potential gene regulatory networks modulated by miRNAs miR-206 and miR-381 in cancer. Our previous analysis showed that myomiRs (including miR-206) and the miRNAs encoded by *DLK1-DIO3* locus (including miR-381) could repress critical pathways implicated in (see Fig 3B and 3C). Therefore, we wanted to investigate *in silico* how miR-206 and miR-381 could regulate molecular pathways in order to suppress tumorigenesis in a pan-cancer manner. Our KEGG pathway analysis through Enrichr revealed that the putative target genes of miR-206 and miR-381 were important players in the development and progression of cancer cells (Fig 6A). This finding was further confirmed by another feature of Enrichr (JENSEN Disease) which highlights the relationship between genes and diseases (Fig 6B).

**Fig 7:**
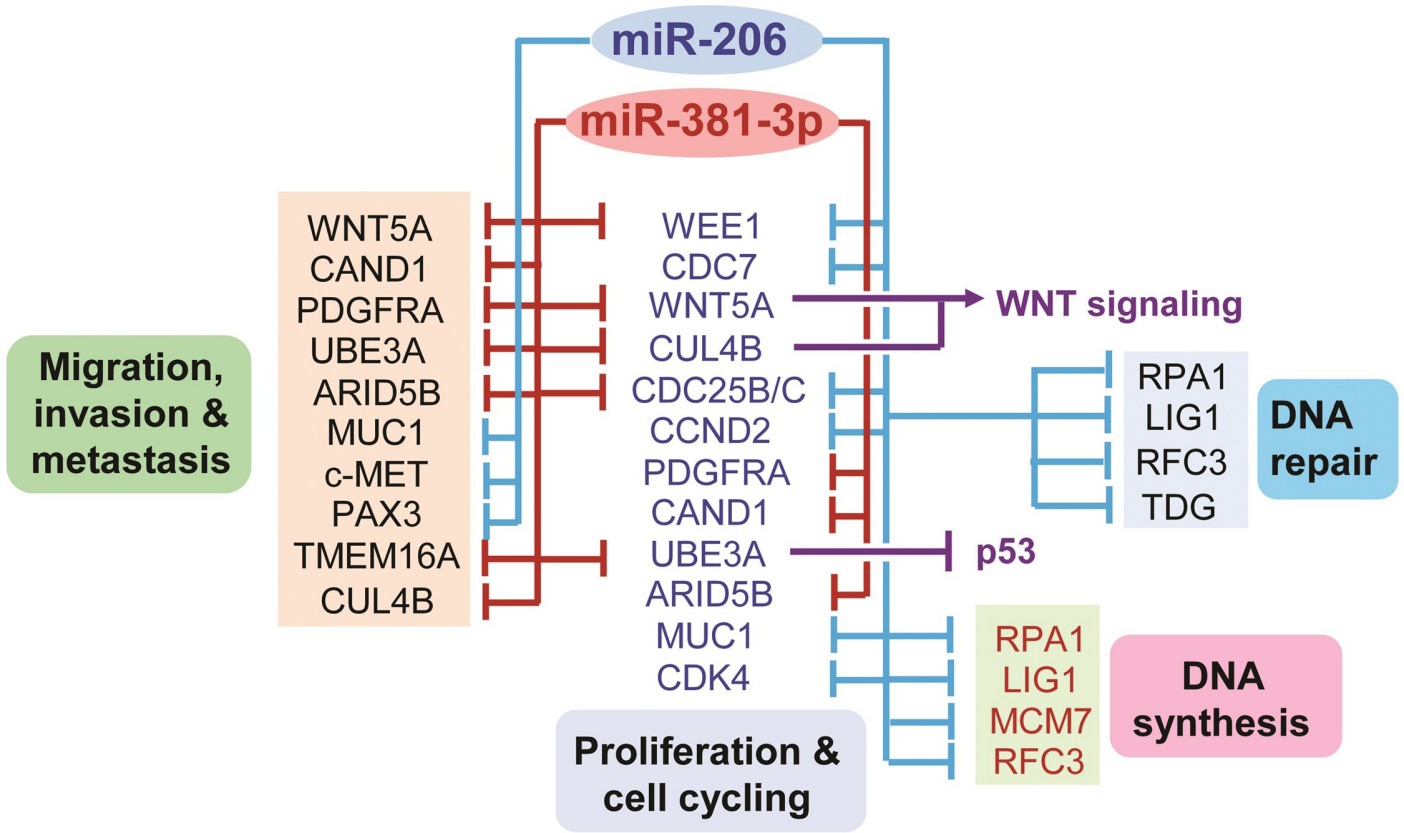
Putative molecular regulatory network mediating the effects of miR-206 and miR-381-3p on carcinogenesis. This diagrams was created based on experimentally validated miRNA/mRNA interactions (obtained from TarBase) within cancer-related processes for miR-206 and miR-381-3p.

We then obtained the list of genes predicted by TargetScan to be co-targeted by both miR-381 and miR-206. This analysis revealed a small set of shared targets (most notably *CCND2*, *ACVR2B*, *NFAT5*, and *CORO1C*) which are known to promote fundamental processes driving tumorigenesis and malignancy [59–69] (S4 Fig). Of note, miR-206 tended to occupy more complementary sites in the 3’ untranslated region (3’ UTR) of its target transcripts than miR-381 (S4 Fig). Since the presence of more miRNA binding sites within the 3’ UTR of target mRNAs leads to a more powerful gene silencing [70, 71], this might at least partially explain why growth-suppressive effects exerted by miR-206 were in most cases more potent than that of miR-381 (see Fig 5B). Finally, we generated a regulatory network using experimentally validated miRNA/mRNA targeting interactions within cancer-related pathways for miR-206 and miR-381 and observed that these two miRNAs can suppress several important aspects of tumorigenesis including DNA replication and repair, cell cycling and proliferation, migration, invasion to neighnoring tissues, and metastatsis to distant parts of the patient’s body (Fig 7). Taken together, our findings highlight key miRNAs which could potentially serve as pan-cancer inhibitors of tumorigenesis in miRNA replacement therapies.

## Conclusion

In this study, we analyzed genome-wide miRNA expression profiles from 14 tumor types and their corresponding normal cell types to obtain miRNAs upregulated in various normal cells compared to cancer cells. We identified 25 miRNAs that were expressed more abundantly in normal cell types compared to their corresponding cancer cell types. Some of these so-called normomiRs had previously been suggested to suppress tumor cell growth, invasion, and metastasis. Moreover, a large fraction of these normomiRs was found to be members of myomiRs and of the largest known miRNA cluster embedded in the *DLK1-DIO3* locus on human chromosome 14. Importantly, myomiRs (*i*.*e*. muscle-enriched miRNAs) as well as miRNAs from the *DLK1-DIO3* locus are frequently reported to be potent suppressors of carcinogenesis. Furthermore, we found that the five-nucleotide CCCGU motif within the miRNA seed region characterized a fraction of tumor-suppressor miRNAs, while simultaneously being depleted from pro-tumor miRNAs. Finally, our functional analysis of nine normomiRs indicated that all the tested normomiRs could suppress at least one cancer cell type *in vitro*. More importantly, we found that two of the select normomiRs, *i*.*e*. miR-206 and miR-381, drastically reduced the viability of five out of six types of cancer cell lines tested, suggesting that these miRNAs might have potent tumor-suppressing impacts in a pan-cancer manner. These findings highlight the potential *in vivo* application of miR-206 (a myomiR) and miR-381 (belonging to the *DLK1-DIO3* miRNAs) in targeting multiple types of tumor. Further investigations using animal models of various cancer types would be needed to determine how normomiRs might function *in vivo*. Taken together, our results suggest that the delivery of miRNAs that are normally expressed at higher levels in various normal cell types but lost in tumor cells (*i*.*e*. normomiR overexpression) might provide an effective approach to miRNA-replacement therapies in multiple cancer types.

## Supporting information

S1 Fig Identification of pan-cancer oncomiRs. (**a**). Heatmap of miRNAs showing higher expression levels in 12 out of 14 cancer types than in corresponding normal cell types. (**b**). The sequence of top 25 pan-cancer oncomiRs identified in our analysis of 14 types of cancer and normal cell types. The nucleotide motifs in blue font indicate the four-nucleotide GUGC motif characteristic of pan-cancer oncomiRs.
(TIF)

S2 Fig The frequency of various short sequence motifs across anti- and pro-tumor miRNAs. (**a**). Table showing the total number of miRNAs with the indicated four-nucleotide sequence motifs across the human miRNome. (**b**). Table showing the total number of miRNAs with the indicated five-nucleotide sequence motifs across the human miRNome. (**c**). Enrichr-based GO analysis of genes predicted to be targeted by the three miRNAs miR-100, miR-99a, and miR-1247. TargetScan, miRDB, and miRanda were used to obtain the predicted targets of the miRNAs.
(TIF)

S3 Fig Effect of miR-381 and miR-206 on the viability of cultured fibroblast cells. Twenty hours after seeding, human dermal fibroblasts were treated with each miRNA, and then subjected to viability assessment using MTS assays three days post-transfection. Ctrl: untreated control; Scr: scrambled control.
(TIF)

S4 Fig Genes predicted to be co-targeted by miR-206 and miR-381. The co-targeted genes of miR-206 and miR-381 were predicted using TargetScan.
(TIF)

S1 Table The list of various experimentally validated oncogenes predicted to be targeted by normomiRs. (XLSX)

S2 Table The frequency of various four-nucleotide sequence motifs observed in oncomiRs or tumor-suppressive miRNAs. (XLS)

S3 Table The frequency of various five-nucleotide sequence motifs observed in oncomiRs or tumor-suppressive miRNAs. (XLS)

S4 Table The putative targets of normomiRs containing the CCCG motif in their seed regions predicted using TargetScan, miRanda, and miRDB. (XLSX)

S5 Table The predicted targets of myomiRs obtained using TargetScan. (XLSX)

S6 Table The predicted targets of the *DLK1-DIO3* locus-embedded miRNAs miR-381, miR-411, and miR-1247 obtained using TargetScan. (XLSX)
