# Supplementary figures and images for: Pan-cancer analysis of microRNA expression profiles highlights microRNAs enriched in normal body cells as effective suppressors of multiple tumor types: A study based on TCGA database
Source: PLoS One. 2022 Apr 27;17(4):e0267291. doi: 10.1371/journal.pone.0267291 (PMC9045663; doi:10.1371/journal.pone.0267291)

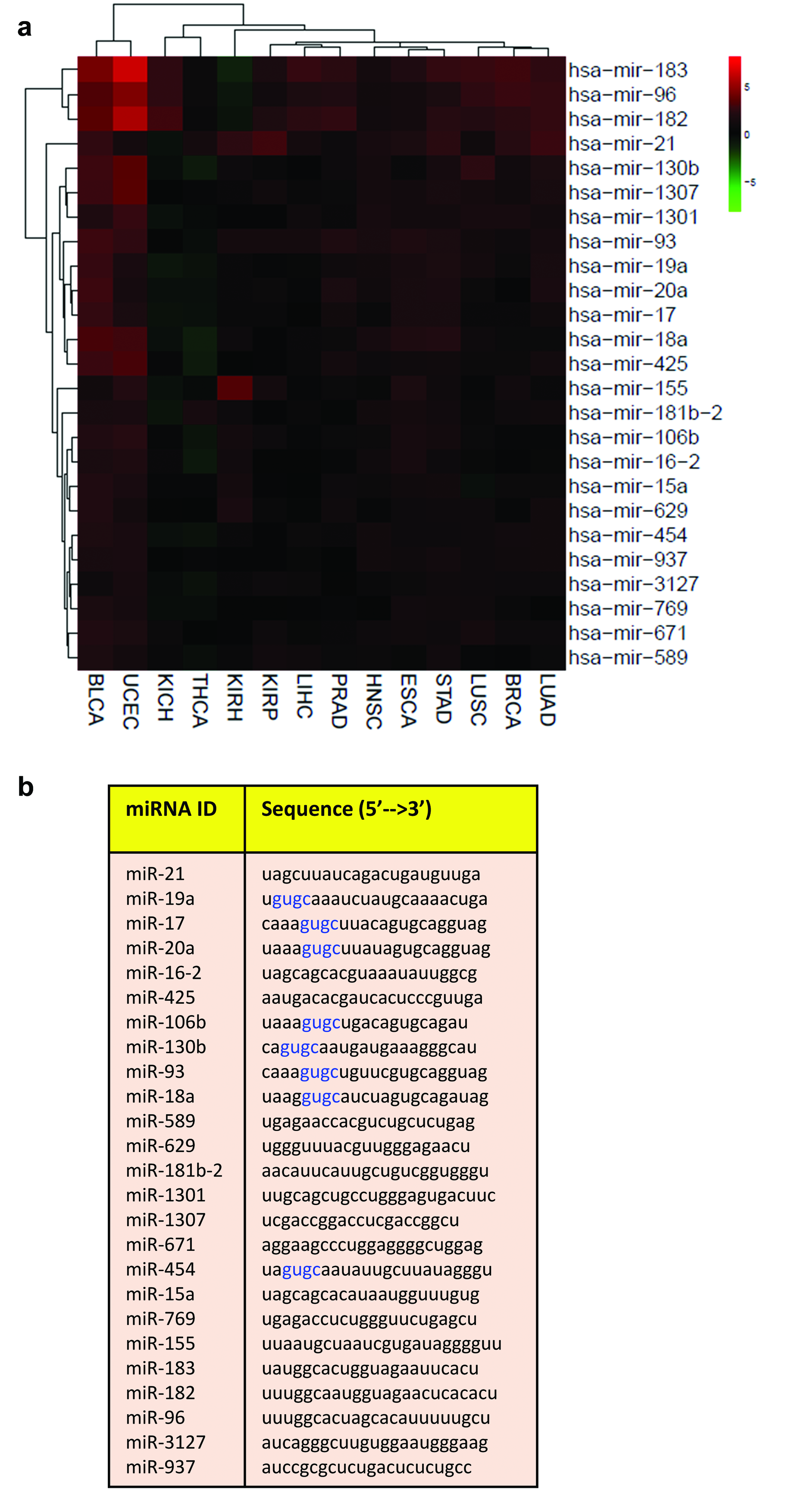

Supplement: S1 Fig — (a). Heatmap of miRNAs showing higher expression levels in 12 out of 14 cancer types than in corresponding normal cell types. (b). The sequence of top 25 pan-cancer oncomiRs identified in our analysis of 14 types of cancer and normal cell types. The nucleotide motifs in blue font indicate the four-nucleotide GUGC motif characteristic of pan-cancer oncomiRs. (TIF) [file pone.0267291.s001.tif]

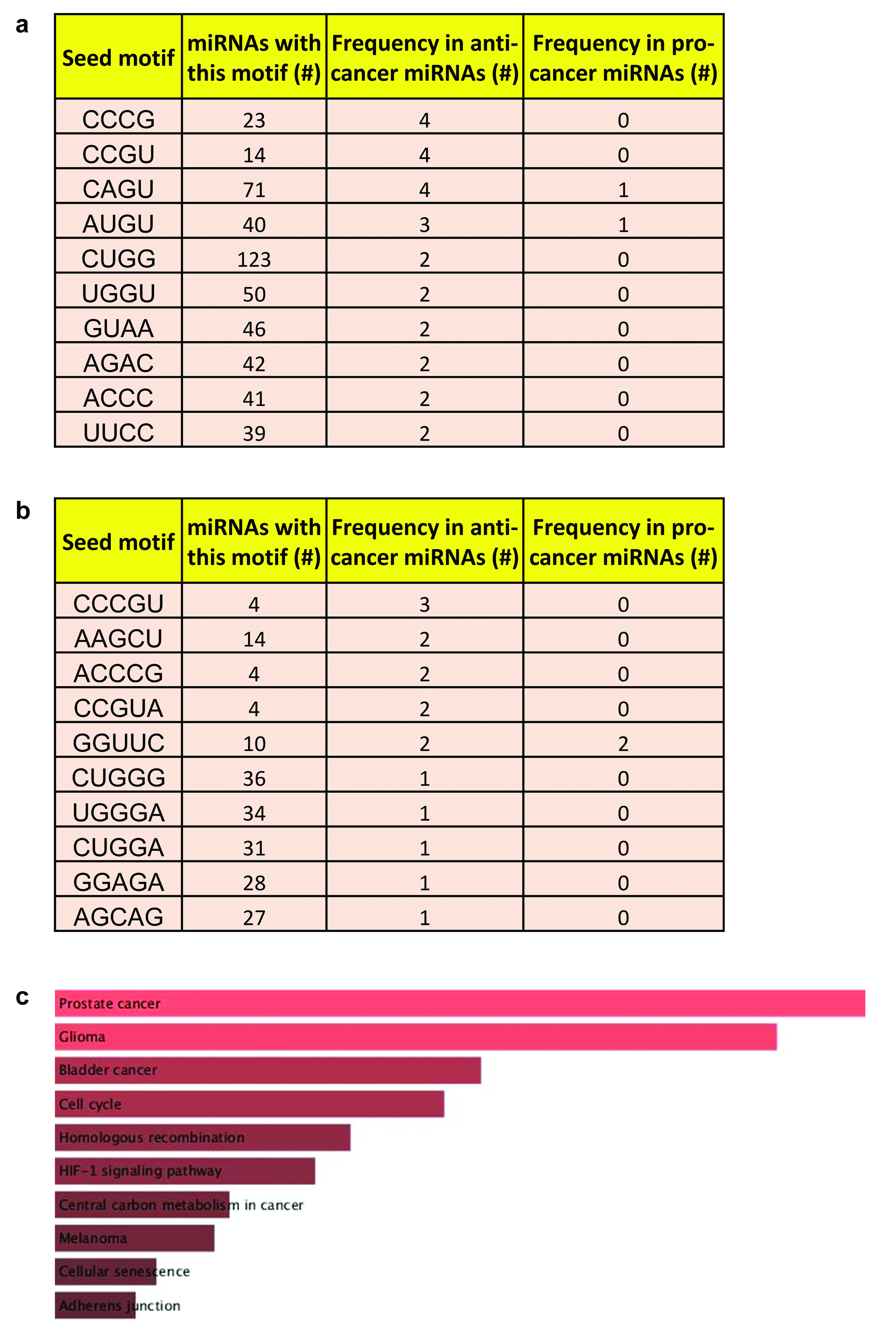

Supplement: S2 Fig — (a). Table showing the total number of miRNAs with the indicated four-nucleotide sequence motifs across the human miRNome. (b). Table showing the total number of miRNAs with the indicated five-nucleotide sequence motifs across the human miRNome. (c). Enrichr-based GO analysis of genes predicted to be targeted by the three miRNAs miR-100, miR-99a, and miR-1247. TargetScan, miRDB, and miRanda were used to obtain the predicted targets of the miRNAs. (TIF) [file pone.0267291.s002.tif]

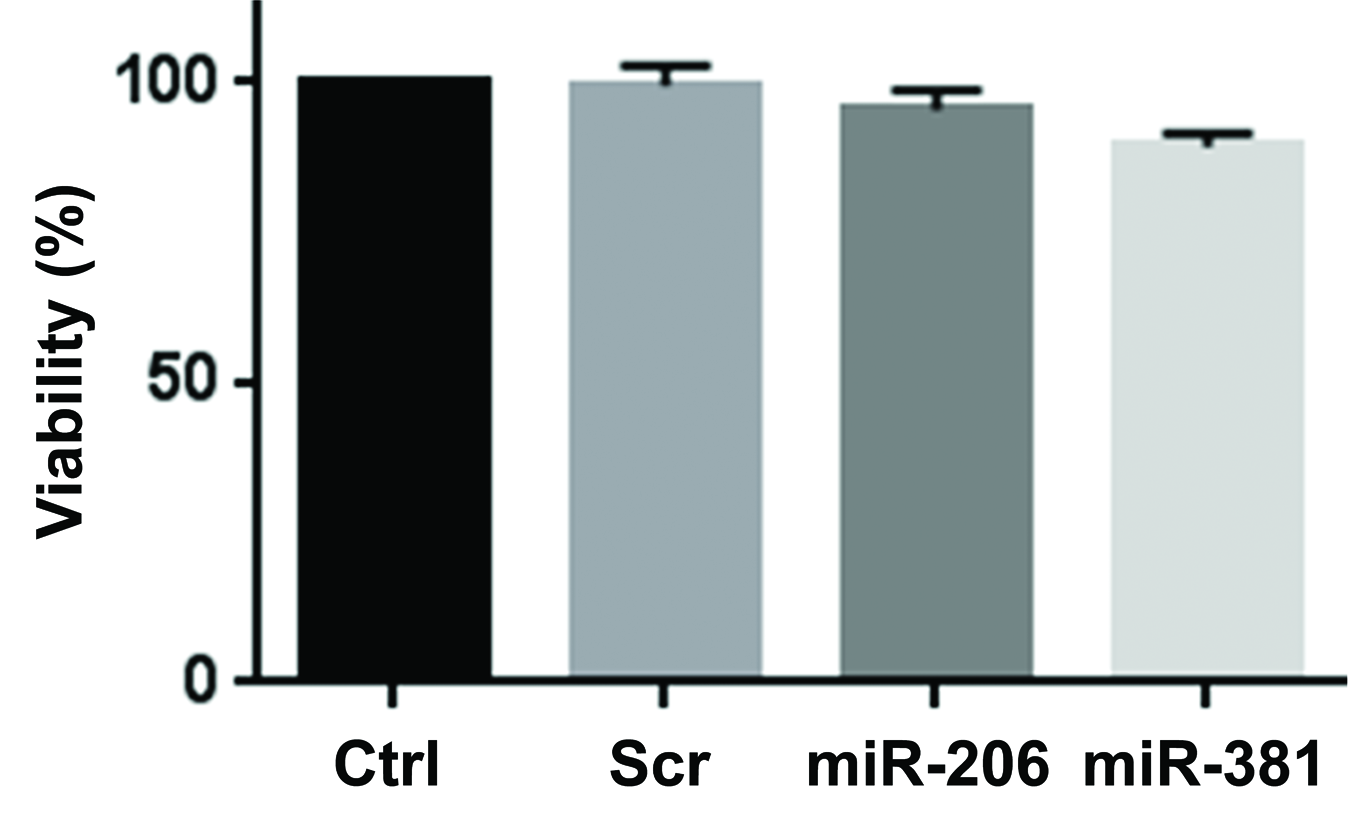

Supplement: S3 Fig — Twenty hours after seeding, human dermal fibroblasts were treated with each miRNA, and then subjected to viability assessment using MTS assays three days post-transfection. Ctrl: untreated control; Scr: scrambled control. (TIF) [file pone.0267291.s003.tif]

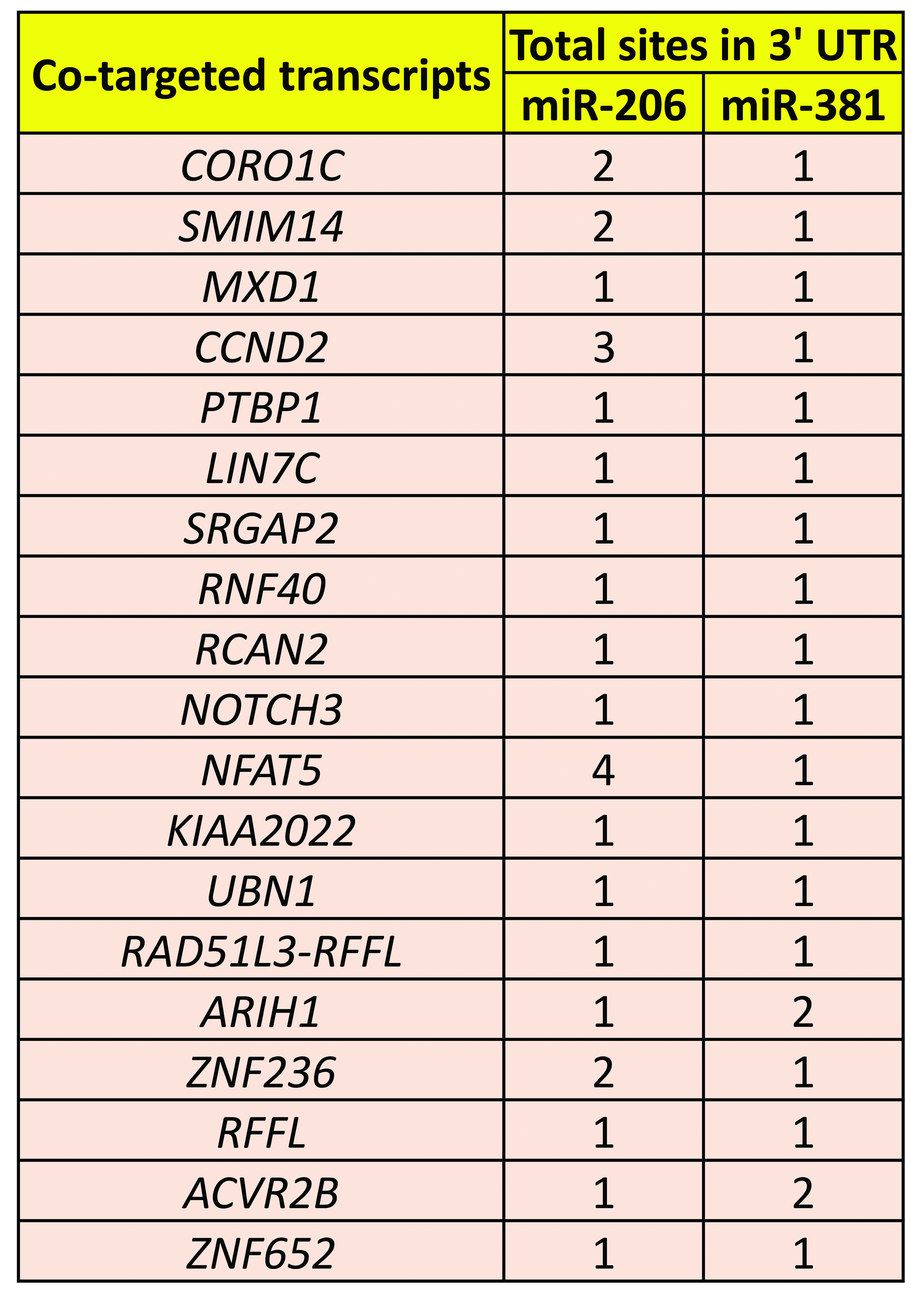

Supplement: S4 Fig — The co-targeted genes of miR-206 and miR-381 were predicted using TargetScan. (TIF) [file pone.0267291.s004.tif]
